# Downregulation of lncRNA XLOC_032768 in diabetic patients predicts the occurrence of diabetic nephropathy

**DOI:** 10.1515/med-2024-0903

**Published:** 2024-03-11

**Authors:** Baohua Li, ZhiLe Wu, Haofeng Xu, HuiLing Ye, Xin Yang

**Affiliations:** Department of General Practice, The First Affiliated Hospital of Guangzhou Medical University, No. 151 Yanjiang West Road, Yuexiu District, Guangzhou, Guangdong, 510030, PR China; Department of Hemodialysis, Guangzhou Guanggang New City Hospital, Guangzhou, Guangdong, 510030, PR China; Department of Geriatrics, The First Affiliated Hospital of Guangzhou Medical University, Guangzhou, Guangdong, 510030, PR China

**Keywords:** XLOC_032768, diabetic nephropathy, type 2 diabetes, diagnosis, prediction

## Abstract

LncRNA XLOC_032768 is reported to prevent renal tubular epithelial cells from cisplatin-induced apoptosis, suggesting its involvement in the development of kidney injury. The present study aimed to explore the role of XLOC_032768 in diabetic nephropathy (DN). The present study enrolled a total of 140 healthy controls (Control group) and 140 patients with type 2 diabetes (Diabetes group). Expression of XLOC_032768 in plasma from these participants was analyzed by performing RT-qPCR. The 140 diabetic patients were followed up for 5 years to monitor the occurrence of diabetic complications. The role of XLOC_032768 in predicting the occurrence of diabetic complications, including DN, diabetic cardiomyopathy (DC), diabetic retinopathy (DR), and diabetic foot (DF) were analyzed by plotting receiver operating characteristic curves and complication-free curves. On the day of admission, plasma levels of XLOC_032768 were not significantly different between Control and Diabetes groups. During follow-up, a total of 22, 15, 13, and 15 cases were diagnosed as DN, DC, DR, and DF, respectively. On the day of diagnosis, plasma levels of XLOC_032768 were only decreased in DN group, but not in other groups, compared to plasma levels of XLOC_032768 on the day of admission. Using plasma levels of XLOC_032768 on the day of admission as a biomarker, potential DN patients were effectively separated from patients with other potential complications and diabetic patients without complications. The 140 diabetic patients were grouped into high and low XLOC_032768 level groups. It was observed that low XLOC_032768 level group showed increased occurrence of DN, but not other complications, compared to high XLOC_032768 level group. Therefore, the downregulation of lncRNA XLOC_032768 in diabetic patients may predict the occurrence of DN.

## 1 Introduction

Diabetes mellitus is a chronic metabolic disorder characterized by elevated blood glucose levels resulting from either insulin deficiency (type 1 diabetes) or insulin resistance (type 2 diabetes) [1–3]. It is a global health concern affecting millions of people worldwide and is associated with various complications that significantly impact patients’ quality of life and overall health outcomes [4,5]. One of the most prevalent and severe complications of diabetes is diabetic nephropathy (DN), a progressive kidney disease that leads to the development of end-stage renal disease [6,7]. DN is characterized by structural and functional changes in the kidneys, including glomerular hypertrophy, glomerular basement membrane thickening, and the accumulation of extracellular matrix proteins in the renal tubules [8–10]. It is estimated that approximately 20–40% of individuals with diabetes develop DN, making it a major cause of morbidity and mortality in diabetic patients [10]. In addition to DN, diabetes can also lead to other microvascular and macrovascular complications, including diabetic cardiomyopathy (DC), diabetic retinopathy (DR), and diabetic foot (DF) [11]. These complications further contribute to the burden of the disease and increase the risk of cardiovascular events, visual impairment, and lower limb amputations.

Identifying reliable biomarkers that can aid in the early detection, prediction, and monitoring of these complications is crucial for implementing appropriate interventions and improving patient outcomes [12,13]. Advances in molecular biology have revealed the regulatory roles of non-coding RNAs, including long non-coding RNAs (lncRNAs), in various physiological and pathological processes [14,15]. LncRNAs are a class of RNA molecules that do not encode proteins but play important roles in gene regulation, chromatin remodeling, and epigenetic modifications. They have been implicated in various diseases, including cancer, cardiovascular diseases, and neurodegenerative disorders [15,16]. LncRNAss are associated with inflammation [17]. On the other hand, type 2 diabetes [18] and diabetic kidney disease [19] are also characterized with increased burden of inflammation. Indeed, many inflammatory and metabolic markers, such as hemogram-derived indices [20], omentin [21], kidney injury molecule [22], prognostic nutritional index [23], and neuregulin [24] are related with diabetes and with diabetic microvascular complications. Thus, studying the association between DN and lncRNAs makes sense. Recent studies have also suggested the involvement of lncRNAs in the pathogenesis of diabetic complications, including DN [25,26].

One lncRNA of particular interest is XLOC_032768, which has been shown to have a protective role in renal tubular epithelial cells. Previous research has demonstrated that XLOC_032768 prevents apoptosis in these cells, suggesting its potential involvement in kidney injury and the development of DN [27,28]. However, the precise mechanisms and clinical implications of XLOC_032768 in diabetic complications, particularly DN, remain largely unknown. Therefore, the present study aimed to explore the role of XLOC_032768 in DN and its potential as a biomarker for predicting the occurrence of DN. A 5-year follow-up analysis showed that the downregulation of lncRNA XLOC_032768 in diabetic patients may predict the occurrence of DN.

## 2 Materials and methods

### 2.1 Patients

A total of 140 healthy controls (81 males and 59 females, 45.1 ± 11.9 years) and 140 patients (81 males and 59 females, 45.4 ± 11.2 years) with type 2 diabetes were enrolled at The First Affiliated Hospital of Guangzhou Medical University from June 2017 to May 2018. The patients were selected based on specific inclusion criteria, including a confirmed diagnosis of type 2 diabetes according to established clinical criteria: a diagnosis of diabetes can be made if A1C levels are equal to or greater than 6.5%. The laboratory test should use a method that is national glycohemoglobin standardization program (NGSP) certified and standardized to the diabetes control and complications trial (DCCT) assay, or fasting plasma glucose levels equal to or greater than 126 mg/dL (7 mmol/L) can also indicate diabetes. Fasting is defined as no caloric intake for at least 8 h, or during an oral glucose tolerance test, if the 2 h plasma glucose level is equal to or greater than 200 mg/dL (11.1 mmol/L), it is considered indicative of diabetes. The test should be performed according to the World Health Organization’s guidelines, using a glucose load containing the equivalent of 75 g anhydrous glucose dissolved in water, or for patients displaying classic symptoms of hyperglycemia or hyperglycemic crisis, a random plasma glucose level equal to or greater than 200 mg/dL (11.1 mmol/L) is indicative of diabetes [29]. Demographic and clinical information, including age, gender, duration of diabetes, and comorbidities, were collected for all participants.


**Ethics approval and consent to participate**: Ethical approval was obtained from the Ethics Committee of The First Affiliated Hospital of Guangzhou Medical University (Approval number: ES-2023-164-01). Written informed consent was obtained from all individual patients included in the study.

### 2.2 Plasma preparation

Peripheral blood samples were collected from all participants under fasting conditions using standard venipuncture techniques. Blood samples were collected in ethylenediaminetetraacetic acid tubes to prevent clotting. Plasma was obtained by centrifuging the blood samples at 1,500 × *g* for 15 min at 4°C. The supernatant plasma was carefully collected, aliquoted, and stored at −80°C until further analysis.

### 2.3 Follow-up

The 140 diabetic patients included in the study were followed up for a period of 5 years. During the follow-up period, the occurrence of diabetic complications, including DN, DC, DR, and DF, was monitored.

### 2.4 qRT-PCR

Total RNA was extracted from plasma samples using RNAzol (Sigma-Aldrich) following the manufacturer’s instructions. The concentration and purity of RNA were assessed using a spectrophotometer. Complementary DNA was synthesized from the extracted RNA using SSRT IV kit (ThermoFisher Scientific) through reverse transcription. qPCR was then performed to measure the expression levels of lncRNA XLOC_032768. The qRT-PCR reactions were performed in triplicate using a SYBR Green-based detection system. The relative expression levels of XLOC_032768 were calculated using the 2^−ΔΔCt^ method, normalized to the reference gene 18S rRNA, and expressed as fold changes compared to control samples.

### 2.5 Statistical analysis

Statistical analysis was performed using GraphPad Prism 6. The differences in XLOC_032768 expression levels between the control and diabetes groups were analyzed using *t*-test because data were normally distributed by Kolmogorov–Smirnov test. Receiver operating characteristic (ROC) curves were generated to evaluate the diagnostic performance of XLOC_032768 in predicting the occurrence of diabetic complications, including DN, DC, DR, and DF. Area under the curve (AUC) values were calculated to assess the discriminatory power of XLOC_032768. Complication-free curves were plotted using Kaplan–Meier analysis, and differences between groups were assessed using log-rank tests. Sample size calculation use normal distribution method and *T*-test method. Statistical significance was set at *p* < 0.05, and all tests were two-tailed.

## 3 Results

### 3.1 Comparison of plasma levels of XLOC_032768 between control and diabetes groups

**Figure 1:**
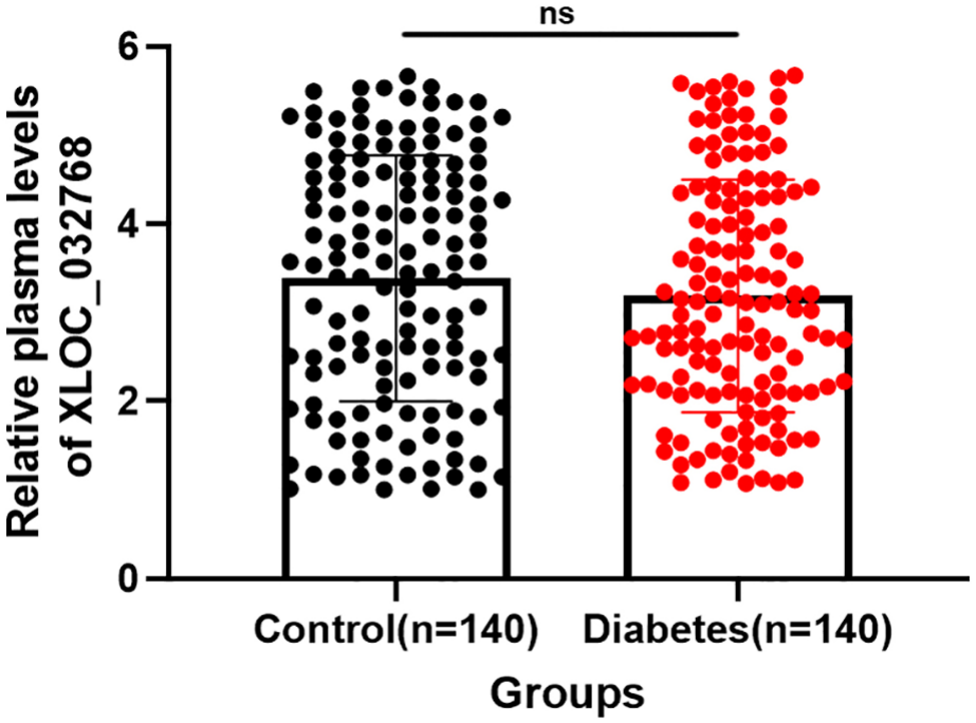
Comparison of plasma levels of XLOC_032768 between control and diabetes group plasma levels of XLOC_032768 in a cohort consisting of 140 healthy controls and 140 patients with type 2 diabetes were determined using qRT-PCR. ns, not statistically significant.

Plasma levels of XLOC_032768 were measured in a cohort of 140 healthy controls and 140 patients with type 2 diabetes. RT-qPCR expression levels of XLOC_032768 in plasma samples were obtained on the day of admission. On the day of admission, there were no significant differences in the plasma levels of XLOC_032768 between the control group and the group of patients with type 2 diabetes (Figure 1, *p* > 0.05). The mean expression levels of XLOC_032768 were comparable between the two groups, indicating that the baseline expression of XLOC_032768 in plasma was similar in individuals without diabetes and those with diabetes. Therefore, the plasma levels of XLOC_032768 did not differ significantly between the control and diabetes groups on the day of admission. This suggests that the initial expression of XLOC_032768 in plasma is not affected by the presence of type 2 diabetes.

### 3.2 Changes in plasma levels of XLOC_032768 in diabetic complications

**Figure 2:**
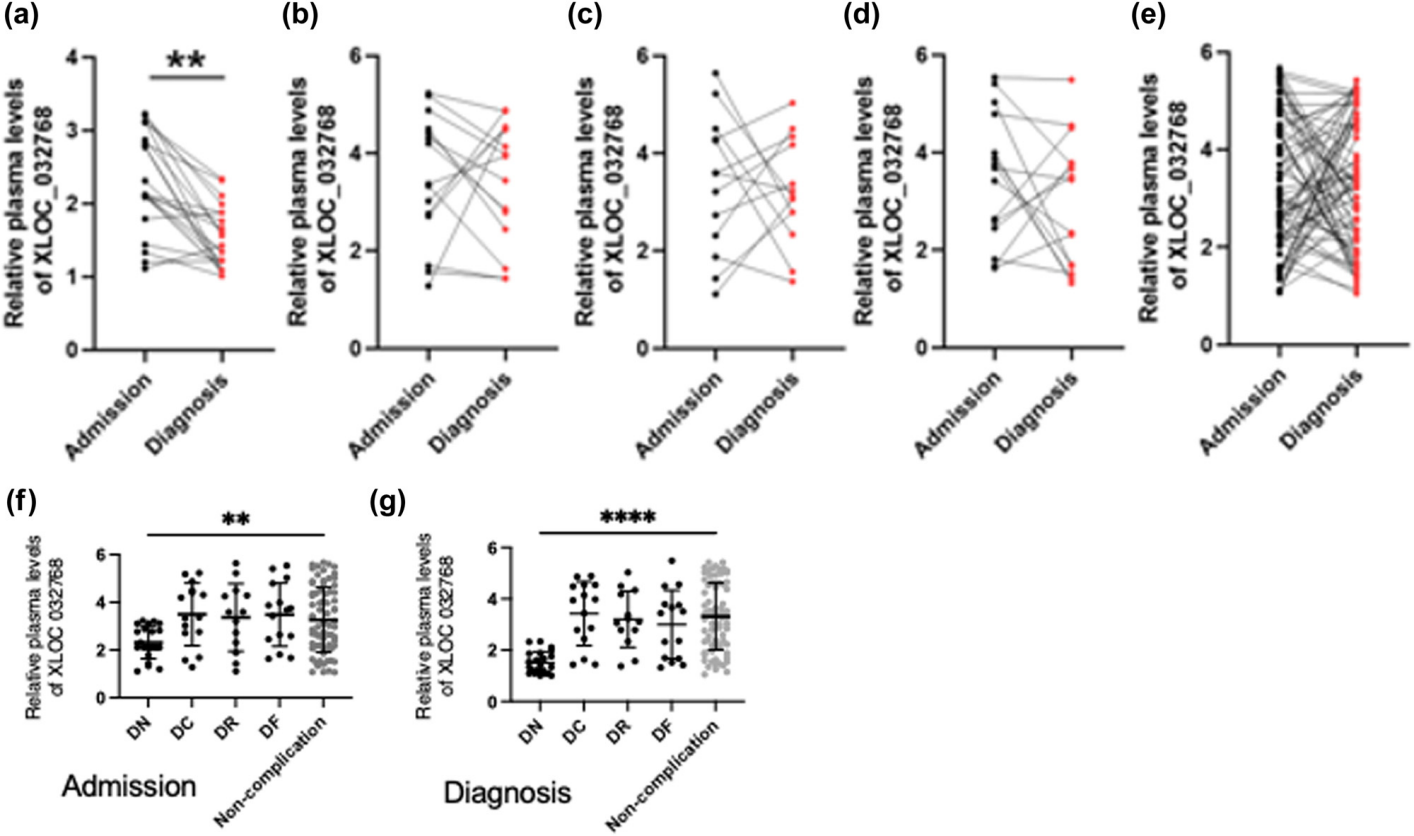
Changes in plasma levels of XLOC_032768 in diabetic complications. During a follow-up period of 5 years, a subset of the 140 diabetic patients developed diabetic complications, including DN, DC, DR, and DF. Plasma samples were collected on the day of diagnosis and compared to the plasma levels of XLOC_032768 obtained on the day of admission in DN (a), DC (b), DR (c), DF (d), and non-complication (e) groups. Compared the significant differences between different groups of admission and diagnosis (f and g). **, *p* < 0.01, ****, *p* < 0.01.

During a follow-up period of 5 years, a subset of the 140 diabetic patients developed diabetic complications, including DN, DC, DR, and DF. Plasma samples were collected on the day of diagnosis and compared to the plasma levels of XLOC_032768 obtained on the day of admission. RT-qPCR was performed to quantify the expression levels of XLOC_032768 in these samples. Among the diabetic patients, a total of 22 cases were diagnosed with DN, 15 with DC, 13 with DR, and 15 with DF. When comparing the plasma levels of XLOC_032768 on the day of diagnosis to those on the day of admission, a significant decrease in XLOC_032768 levels was observed specifically in the DN group (Figure 2a, *p* < 0.05). In contrast, no significant differences in XLOC_032768 levels were detected in DC (Figure 2b), DR (Figure 2c), and DF (Figure 2d) diabetic complication groups as well as the non-complication group (Figure 2e) compared to their respective levels on the day of admission (*p* > 0.05). Therefore, the plasma levels of XLOC_032768 were significantly decreased specifically in the DN group, indicating its potential involvement in the development and progression of DN. We also have compared the significant differences between different groups of admission and diagnosis. Surprisingly, we discovered that XLOC_032768 expression level is decreased in DN groups than in non-complication group in admission and diagnosis (Figure 2f and g) and further decreased in diagnosis as shown in Figure 2a, suggesting that the downregulation of XLOC_032768 may be specifically associated with the pathogenesis of DN.

### 3.3 Use of plasma levels of XLOC_032768 as a biomarker for DN

**Figure 3:**
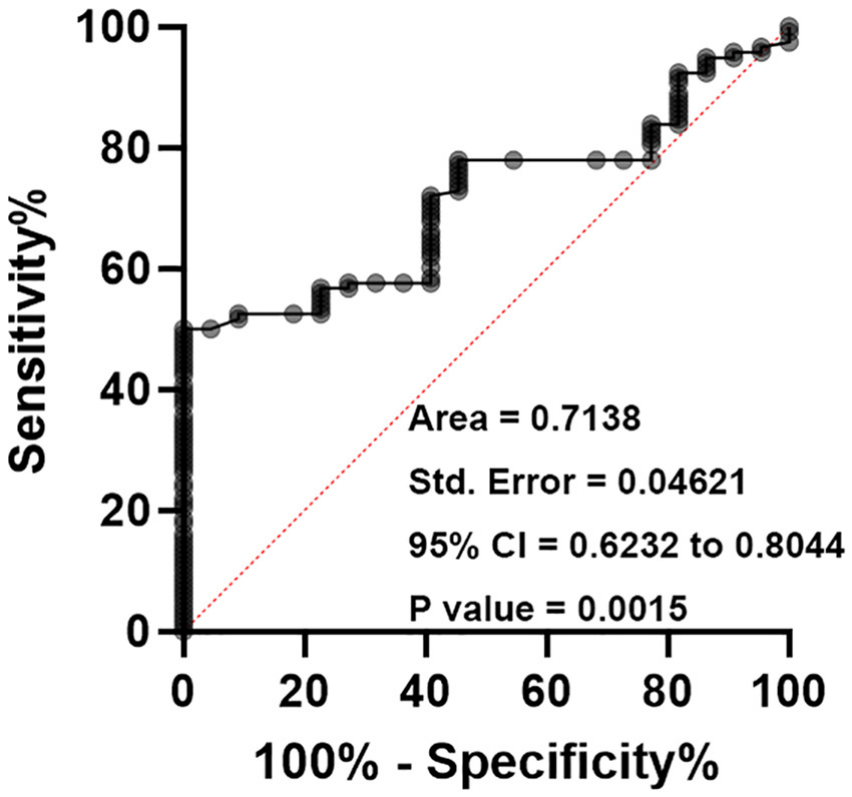
Use of plasma levels of XLOC_032768 as a biomarker for DN in order to evaluate the diagnostic potential of plasma levels of XLOC_032768 in predicting the occurrence of DN, we conducted ROC curve analysis. In this analysis, DN patients were classified as true positive cases, while other diabetic patients without DN were classified as true negative cases.

To assess the diagnostic potential of plasma levels of XLOC_032768 for predicting the occurrence of DN, ROC curve analysis was performed. DN patients were considered as true positive cases, and other diabetic patients without DN were considered as true negative cases. ROC curve analysis revealed that plasma levels of XLOC_032768 on the day of admission effectively separated potential DN patients from patients with other potential complications and diabetic patients without complications (Figure 3). The AUC (>0.70) value is big, and the sensitivity is high, conversely, specificity is low indicating a good discriminatory power of XLOC_032768 as a biomarker for DN. Therefore, plasma levels of XLOC_032768 on the day of admission hold promise as a potential biomarker for predicting the occurrence of nephropathy (DN). The ability of XLOC_032768 can effectively differentiate DN patients from individuals with other complications.

### 3.4 Association between XLOC_032768 levels and DN occurrence

**Figure 4:**
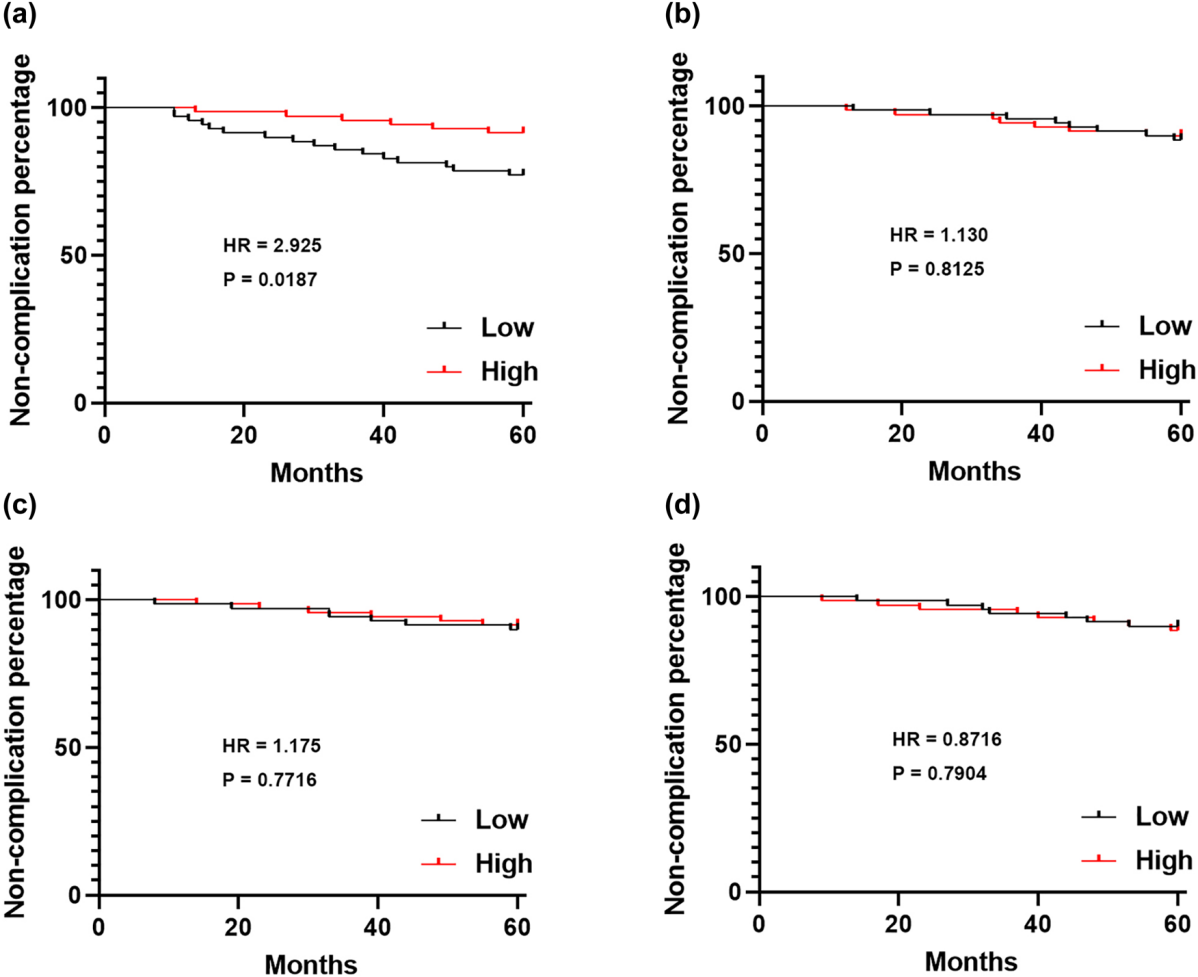
Association between XLOC_032768 levels and DN occurrence in the 140 diabetic patients were divided into two groups based on their plasma levels of XLOC_032768: high XLOC_032768 level group and low XLOC_032768 level group (*n* = 40). Complication-free curve analysis was performed to assess the relationship between XLOC_032768 levels and the occurrence of DN (a), DC (b), DR (c), and DF (d).

The 140 diabetic patients were divided into two groups based on their plasma levels of XLOC_032768: high XLOC_032768 level group and low XLOC_032768 level group. According to the plasma average, levels of XLOC_032768 were measured in a cohort of 140 healthy controls, comparing with 140 patients with type 2 diabetes to divide the groups as low and high expression. Complication-free curve analysis was performed to assess the relationship between XLOC_032768 levels and the occurrence of DN, as well as other diabetic complications. The complication-free curve analysis demonstrated that the low XLOC_032768 level group exhibited a higher incidence of DN compared to the high XLOC_032768 level group (Figure 4a). However, no significant differences in the occurrence of other diabetic complications, including DC (Figure 4b), DR (Figure 4c), and DF (Figure 4d), were observed between the two groups. The results indicate that decreased levels of XLOC_032768 in plasma are specifically associated with an increased risk of developing DN in diabetic patients.

## 4 Discussion

The results of the present study provide important insights into the potential role of lncRNA XLOC_032768 as a predictive biomarker for DN in patients with type 2 diabetes. The findings demonstrate that the downregulation of XLOC_032768 in plasma samples is associated with the occurrence of DN, while its levels remain relatively stable in patients without DN and in individuals from the control group.

The observed downregulation of XLOC_032768 specifically in the DN group in admission and further decrease in diagnosis than in non-complication group, suggests its involvement in the pathogenesis of DN. This finding is consistent with previous studies that have demonstrated the protective role of XLOC_032768 in renal tubular epithelial cells against apoptosis [27,28]. It is plausible that the reduced expression of XLOC_032768 may disrupt the protective mechanisms, leading to increased susceptibility to renal damage and the development of DN. The ability of XLOC_032768 levels on the day of admission to effectively distinguish potential DN patients from those with other potential complications and diabetic patients without complications indicates its potential utility as a predictive biomarker. The use of ROC curves and complication-free curves allowed for the evaluation of XLOC_032768 as a diagnostic tool for DN. This suggests that assessing XLOC_032768 expression levels at an early stage may aid in identifying individuals at higher risk of developing DN, enabling timely intervention and management strategies to prevent or delay the progression of this complication.

Comparing these findings to previous studies, the results of the present study align with emerging evidence that highlights the dysregulation of lncRNAs in the context of diabetic complications [15,25]. Several studies have reported altered expression patterns of lncRNAs in DN, emphasizing their potential as biomarkers and therapeutic targets. The downregulation of XLOC_032768 in the DN group further strengthens the growing body of evidence implicating lncRNAs in the pathogenesis of DN [30,31]. However, it is important to acknowledge some limitations of the present study. First, the sample size was relatively small, which may limit the generalizability of the findings. Larger cohorts involving diverse populations are needed to validate the results and establish the clinical utility of XLOC_032768 as a biomarker for DN. Additionally, the follow-up period of 5 years might not capture the entire spectrum of DN progression, and longer-term studies are warranted to assess the durability of XLOC_032768 as a predictive biomarker. Further research is also required to elucidate the underlying molecular mechanisms through which XLOC_032768 contributes to the development and progression of DN. Investigating the interactions between XLOC_032768 and key genes or signaling pathways involved in renal pathology could provide valuable insights into its functional role and potential therapeutic implications. Additionally, exploring the potential interplay between XLOC_032768 and other lncRNAs or regulatory molecules associated with DN could shed light on the complex network of non-coding RNAs involved in this diabetic complication.

In conclusion, the findings of this study support the downregulation of lncRNA XLOC_032768 in diabetic patients as a potential predictive biomarker for the occurrence of DN. The results highlight the importance of the role of lncRNAs in the pathogenesis of diabetic complications and emphasize the potential of XLOC_032768 as a diagnostic tool for identifying individuals at higher risk of developing DN. Future studies should focus on validating these findings in larger cohorts, exploring the underlying molecular mechanisms to advance our understanding of the role of XLOC_032768 in DN and other diabetic complications, and determining whether DN can be treated by XLOC_032768 supplementation.
